# Patterns of Glomerular Injury: Histopathological Classification and Clinical Correlation

**DOI:** 10.7759/cureus.91728

**Published:** 2025-09-06

**Authors:** Hussein Qasim, Mohammad Abu Shugaer, Shaima' Dibian, Mahfouz Ktaifan, Karis Khattab, Matteo Luigi Giuseppe Leoni, Giustino Varrassi

**Affiliations:** 1 Department of Pathology and Laboratory Medicine, Jordan University of Science and Technology, Irbid, JOR; 2 Department of Medicine, College of Medicine and Health Sciences, An-Najah National University, Nablus, PSE; 3 Faculty of Medicine, Jordan University of Science and Technology, Irbid, JOR; 4 Department of Medical and Surgical Sciences and Translational Medicine, Sapienza University, Rome, ITA; 5 Department of Pain Medicine, Fondazione Paolo Procacci, Rome, ITA

**Keywords:** glomerular disease, histopathology, immunofluorescence, nephritic syndrome, nephrotic syndrome, renal biopsy

## Abstract

Glomerular diseases represent a diverse group of conditions that significantly contribute to chronic kidney disease (CKD) and end-stage renal disease (ESRD) worldwide. Understanding the histopathological patterns of glomerular injury is essential for accurate diagnosis, prognosis, and targeted therapy. This comprehensive review explores the morphologic, immunopathologic, and clinical characteristics of major glomerular disorders, including minimal change disease, focal segmental glomerulosclerosis, membranous nephropathy, immunoglobulin A nephropathy, lupus nephritis, and others. Histological classification is integrated with clinical syndromes, such as nephrotic and nephritic presentations, emphasizing the role of light microscopy, immunofluorescence, and electron microscopy in renal biopsy interpretation. Recent advances in molecular diagnostics, digital pathology, and artificial intelligence are also discussed, highlighting their transformative impact on precision nephrology. Through a multidisciplinary lens, this review underscores the prognostic and therapeutic implications of distinct histologic patterns and advocates for the continued evolution of clinicopathologic correlation in glomerular disease management.

## Introduction and background

The glomerulus serves as the primary filtration unit of the kidney, structurally comprising a specialized tuft of capillaries encased within Bowman’s capsule [1]. Its filtration barrier, also known as the glomerular filtration barrier (GFB), is a highly selective tri-layered interface formed by fenestrated endothelial cells, the glomerular basement membrane (GBM), and visceral epithelial cells (podocytes) [2]. Together, these components enable the ultrafiltration of plasma, restricting the passage of large macromolecules and negatively charged proteins, while allowing water and small solutes to pass into the urinary space [3]. This sophisticated barrier maintains plasma oncotic pressure, prevents proteinuria under normal conditions, and plays a pivotal role in maintaining systemic fluid and electrolyte balance [4]. The integrity of the glomerular filtration apparatus is critical to renal function [5]. Disruption of this barrier, whether due to immune complex deposition, podocyte injury, basement membrane abnormalities, or endothelial dysfunction, leads to glomerular injury, which is a hallmark of many primary and secondary kidney diseases [6]. Among these, glomerular diseases are a major contributor to chronic kidney disease (CKD) and end-stage renal disease (ESRD) globally, imposing a significant burden on healthcare systems and patient quality of life [7]. Notably, podocyte injury, either through structural damage, detachment, or effacement of foot processes, has emerged as a central mechanism in many proteinuric disorders [8]. Clinically, glomerular diseases present along a spectrum, most commonly as nephrotic syndrome, nephritic syndrome, or asymptomatic urinary abnormalities, depending on the underlying pathophysiology [9]. Nephrotic syndrome is characterized by heavy proteinuria, hypoalbuminemia, and edema and is often seen in diseases with podocyte dysfunction, such as minimal change disease and membranous nephropathy (MN) [10]. In contrast, nephritic syndrome features hematuria, hypertension, and reduced glomerular filtration and is more typical of inflammatory or proliferative glomerulonephritides such as IgA nephropathy and lupus nephritis [10]. Accurate diagnosis and classification of glomerular diseases depend heavily on histopathological evaluation, typically through renal biopsy, which provides critical insights into disease etiology, activity, chronicity, and potential reversibility [11]. The integration of light microscopy (LM), immunofluorescence (IF), and electron microscopy (EM) findings allows renal pathologists to distinguish between various morphologic and immunopathologic patterns of injury [12]. These findings not only aid in establishing an etiologic diagnosis but also have significant implications for treatment strategies and prognosis [13]. In this review, we aim to describe the patterns of glomerular injury, emphasizing histopathological classification, clinical correlation, and the implications for prognosis and therapy.

## Review

Methodology

This is a narrative review designed to synthesize and critically appraise current evidence on the histopathological patterns of glomerular injury and their clinical correlations. The review followed the Scale for the Assessment of Narrative Review Articles (SANRA) to ensure rigor and transparency. A comprehensive literature search was conducted across PubMed, Scopus, Web of Science, Embase, and Google Scholar, using Medical Subject Headings (MeSH) and keywords such as "glomerulonephritis", "renal biopsy", "histopathology", "focal segmental glomerulosclerosis", "lupus nephritis", and "IgA nephropathy". Boolean operators were applied to refine search results, and backward citation tracking was used to identify additional relevant publications. Inclusion criteria consisted of peer-reviewed original studies and review articles discussing glomerular pathology, histologic classification, clinicopathologic correlation, or diagnostic modalities (IF and EM). Exclusion criteria included editorials, case reports, and abstracts, unless conceptually significant. Screening was performed independently by two reviewers, with disagreements resolved by consensus with a third reviewer. This approach prioritizes a descriptive thematic synthesis rather than a quantitative meta-analysis; therefore, no pooled statistical analysis or formal risk-of-bias scoring was performed, in line with the scope of a narrative review. Potential selection bias inherent to narrative reviews is acknowledged, and emphasis was placed on citing seminal studies, recent guideline updates, and landmark clinical trials to ensure clinical relevance.

Glomerular pathophysiology

Most glomerulonephritides are immune-mediated, resulting from complex interactions between circulating immune elements and intrinsic glomerular cells [14]. Immune complex deposition is a common mechanism: antigen-antibody complexes deposit in glomeruli (or form in situ) and incite injury via complement activation, recruitment of leukocytes, and release of inflammatory mediators [15]. The site of immune deposits influences the pattern of damage; for example, subepithelial deposits (between podocytes and the GBM) typically cause membranous changes, whereas subendothelial or mesangial deposits provoke endocapillary inflammation and proliferative changes [16]. Complement dysregulation can also drive glomerular injury, even without abundant immunoglobulin deposition, through uncontrolled activation of complement pathways on the capillary wall [17]. Podocyte injury is another key pathogenic process, often underpinning proteinuric diseases [18]. Podocytes are terminally differentiated cells critical for GFB integrity; when injured by immune attack or other insults, they detach or lose their slit diaphragm function, leading to heavy proteinuria [19]. Diseases such as minimal change disease (MCD) and focal segmental glomerulosclerosis (FSGS) are considered “podocytopathies,” unified by podocyte foot process effacement and cytoskeletal disruption [20]. Endothelial cell injury and microvascular thrombosis, such as in thrombotic microangiopathies, represent another pattern, resulting in capillary wall necrosis and fibrin deposition [21]. Severe glomerular injuries often induce proliferation of parietal epithelial cells and fibrin leakage, forming crescents (extracapillary cellular accumulations) that signify rapidly progressive glomerulonephritis (RPGN) [22].

Histopathological classification of glomerular diseases

Histopathologic classification of glomerular diseases can be approached along three major axes: (1) morphological patterns of injury observed by LM, (2) etiology - whether primary (kidney-limited) or secondary to a systemic process, and (3) immunopathologic mechanisms (as determined by IF and serological context) [23]. Modern pathology reports integrate these aspects to reach a comprehensive diagnosis [24]. Table 1 summarizes the major histologic patterns of glomerular injury, with typical features and examples.

**Table 1:** Comprehensive summary of key histopathological patterns of glomerular injury based on light microscopy, immunofluorescence, and electron microscopy findings, with primary and secondary etiologies. GN: Glomerulonephritis; RPGN: Rapidly Progressive GN; GBM: Glomerular Basement Membrane; IFTA: Interstitial Fibrosis and Tubular Atrophy; IF: Immunofluorescence; EM: Electron Microscopy; LM: Light Microscopy; LCDD: Light Chain Deposition Disease; ANCA: Anti-Neutrophil Cytoplasmic Antibody

Pattern	Light Microscopy	IF/EM Findings	Etiology/Associated Conditions
Minimal Change Disease (MCD) [25]	Normal glomeruli; foamy tubules possible	IF: negative; EM: diffuse podocyte foot process effacement	Primary (idiopathic, especially in children); Secondary: NSAIDs, lithium, rifampin, interferon therapy, Hodgkin lymphoma, other lymphoproliferative disorders, allergic reactions, post-vaccination
FSGS [26,27]	Segmental sclerosis in some glomeruli	IF: ±IgM/C3 in sclerotic areas; EM: patchy foot process effacement	Primary (idiopathic, circulating permeability factors); Secondary: APOL1 risk alleles (African descent), obesity, reflux nephropathy, HIV, heroin, adaptive responses (solitary kidney, reduced nephron mass), hypertension, drug-induced (pamidronate, interferon), familial mutations (NPHS2, ACTN4, TRPC6)
Membranous Nephropathy (MN) [28]	Diffuse capillary wall thickening, GBM spikes	IF: granular IgG/C3; EM: subepithelial deposits	Primary: PLA2R, THSD7A, NELL-1, Sema3B autoantibodies; Secondary: Lupus nephritis (class V), hepatitis B/C, syphilis, solid tumors, drugs (gold, penicillamine, NSAIDs), sarcoidosis
MPGN [29]	Mesangial + endocapillary proliferation, GBM double contours	IF: IgG ± C3 (immune-complex type); dominant C3 (complement-mediated); or dominant C4 (rare); EM: Subendothelial or intramembranous deposits	Immune–complex mediated: Infections (HCV—mixed cryoglobulinemia, HBV, bacterial endocarditis, shunt nephritis, chronic abscess/osteomyelitis, infected prostheses/AV grafts, tuberculosis, malaria, schistosomiasis); Autoimmune/systemic (SLE, Sjögren’s, rheumatoid arthritis, mixed connective-tissue disease, cryoglobulinemic vasculitis types II/III); Monoclonal Ig–related (PGNMID—often IgG3κ, type I cryoglobulinemia, MGRS/MGUS, multiple myeloma, CLL/Waldenström). Complement-mediated: C3 glomerulopathy (C3GN, dense deposit disease) from alternative pathway dysregulation—CFH/CFI/CFB/C3 variants, CFHR5 nephropathy, factor H autoantibodies, C3 nephritic factor; C4 glomerulopathy (rare) from classical/lectin pathway dysregulation—C4 nephritic factor, C1q/C2/C4 variants, lectin pathway defects (MBL/MASP2). MPGN-like pattern without immune/complement deposition (chronic endothelial injury): Thrombotic microangiopathy (aHUS, Shiga-toxin HUS), malignant hypertension, drug-induced TMA (calcineurin inhibitors, VEGF inhibitors, gemcitabine, mitomycin-C), antiphospholipid syndrome, scleroderma renal crisis, radiation nephritis, transplant glomerulopathy (chronic antibody-mediated injury).
Mesangial Proliferative GN [30]	Mesangial hypercellularity	IF: dominant IgA (±C3); EM: mesangial deposits	Primary IgA nephropathy (Berger disease). Secondary causes include IgA vasculitis (Henoch–Schönlein purpura), chronic liver disease (especially cirrhosis), gastrointestinal disorders such as celiac disease and inflammatory bowel disease, autoimmune diseases (including systemic lupus erythematosus, rheumatoid arthritis, and Sjögren’s syndrome), chronic infections (HIV, hepatitis B and C, tuberculosis, parasitic infections), pulmonary conditions (bronchiectasis, cystic fibrosis, chronic lung infections), dermatologic disorders (psoriasis, dermatitis herpetiformis), and systemic conditions such as ankylosing spondylitis, reactive arthritis, as well as recurrence in kidney allografts.
Crescentic GN (RPGN) [31]	Crescents in Bowman’s space, fibrinoid necrosis	IF varies: pauci-immune (ANCA), linear (anti-GBM), granular (immune-complex); EM: GBM rupture	ANCA-associated vasculitis (GPA, MPA, EGPA), anti-GBM disease (Goodpasture), lupus nephritis, post-infectious GN, IgA nephropathy, cryoglobulinemia, endocarditis-associated GN
Chronic Sclerosing GN [32]	Global glomerulosclerosis, IFTA	IF: nonspecific or negative; EM: GBM collapse, no active deposits	End-stage kidney disease of any cause: diabetic nephropathy, hypertensive nephrosclerosis, chronic GN, reflux nephropathy, advanced systemic disease, aging
Nodular Glomerulosclerosis [33]	Mesangial nodules (Kimmelstiel–Wilson); hyaline arterioles	IF: linear IgG (diabetes) or monoclonal light chains; Congo red in amyloid; EM: thick GBM ± fibrils	Diabetic nephropathy (classic), amyloidosis (AL, AA, hereditary), light chain deposition disease, fibrillary GN, immunotactoid GN, monoclonal gammopathy of renal significance

Morphologic patterns in glomerular disease are descriptive and not diagnostic on their own [34]. Terms such as “membranoproliferative” or “crescentic” describe patterns of injury that may arise from diverse causes, including autoimmune, infectious, or complement-mediated processes [35]. Consequently, accurate diagnosis depends on integrating morphology, IF findings, and clinical context [36]. A single disease can display multiple histologic forms; for example, lupus nephritis may range from mesangial to proliferative to membranous forms, and immunoglobulin A (IgA) nephropathy can vary from mild mesangial changes to aggressive crescent formation [37]. Glomerular diseases are generally classified as primary or secondary [38,39]. Primary glomerulopathies are intrinsic to the kidney with no systemic cause, such as MCD, primary FSGS, idiopathic MN, and classic IgA nephropathy [38]. Secondary forms are associated with systemic diseases such as lupus, diabetes, infections, or plasma cell disorders [40]. Recognizing secondary causes is critical, as treatment targets the underlying disease. Morphologic clues often suggest a systemic origin, for example, nodular glomerulosclerosis in diabetic nephropathy or a full-house IF pattern in lupus [33]. Immunopathologic classification further refines diagnosis, grouping glomerulonephritides by the type and pattern of immune deposits [41]. These include immune complex-mediated GN (e.g., lupus nephritis, IgA nephropathy), pauci-immune GN (typically ANCA-associated vasculitis), anti-GBM disease, monoclonal immunoglobulin deposition diseases (e.g., proliferative glomerulonephritis with monoclonal immunoglobulin deposits (PGNMID) or light chain deposition disease (LCDD)), and C3 glomerulopathy [42]. The deposit location also helps correlate with clinical syndrome: subepithelial in MN is often nephrotic, while subendothelial in lupus signals inflammation and hematuria [43]. Pauci-immune GN shows minimal immune deposits and is linked to anti-neutrophil cytoplasmic autoantibody (ANCA)-associated vasculitis, leading to rapidly progressive, necrotizing lesions [31]. Anti-GBM disease involves linear IgG staining due to antibodies against type IV collagen, often presenting with both kidney and lung involvement, as in Goodpasture’s syndrome [44]. Monoclonal immunoglobulin deposition diseases involve restricted deposition of a single light or heavy chain, commonly tied to plasma cell disorders [45]. C3 glomerulopathy, caused by alternative complement pathway dysregulation, features dominant C3 staining with minimal immunoglobulin and frequently shows a membranoproliferative pattern on microscopy [17]. To streamline classification, the Mayo Clinic and Renal Pathology Society proposed a framework in 2016 that groups GN into five major categories: immune complex-mediated, pauci-immune, anti-GBM, monoclonal immunoglobulin-associated, and C3 glomerulopathy [46]. This system emphasizes underlying mechanisms and often aligns with clinical and etiologic classifications [46]. Comprehensive biopsy reports include the disease name, dominant histologic pattern, and immunopathologic category, with mention of coexisting lesions such as crescents or sclerosis, which carry important prognostic weight [47].

Clinical correlation: nephrotic vs. nephritic syndromes

Glomerular diseases classically present with one of several clinical syndromes depending on the nature and severity of injury [48]. The two main presentations are nephrotic syndrome and nephritic syndrome, which represent ends of a clinical spectrum, although overlap is frequently seen [48]. Nephrotic syndrome is defined by heavy proteinuria (>3.5 g/day), hypoalbuminemia, edema, and often hyperlipidemia [49]. Urine sediment is typically bland with few cells or casts [50]. This syndrome results from podocyte or GBM injury without significant inflammation, allowing protein to leak into the urine [8]. Edema, often facial or periorbital in the morning and dependent later, is due to decreased oncotic pressure [51]. The liver compensates for hypoalbuminemia by increasing lipoprotein production, leading to hypercholesterolemia and lipiduria (fatty casts, oval fat bodies) [49]. Common causes include MCD, primary FSGS, MN, and advanced diabetic nephropathy [49]. Patients often lack gross hematuria or significant hypertension [49]. Complications include thromboembolism from loss of anticoagulants such as antithrombin III and infections due to immunoglobulin loss [52]. Nephritic syndrome, by contrast, is marked by hematuria (often gross and cola-colored), RBC casts, hypertension, mild-to-moderate edema, and variable proteinuria usually below the nephrotic range [53,54]. Oliguria and rising creatinine reflect decreased filtration due to active inflammation [55]. It indicates glomerular capillary wall damage and immune complex deposition with complement activation [55]. Common causes include post-infectious GN, IgA nephropathy (especially during flares), and proliferative lupus nephritis [55]. Symptoms often follow triggers such as infections [55]. Urinalysis typically shows dysmorphic RBCs and red cell casts, which indicate glomerular bleeding [56]. Severe cases may evolve into RPGN, featuring crescents on biopsy and rapid renal decline [57]. RPGN is a medical emergency and is commonly caused by ANCA-associated GN, anti-GBM disease, or severe immune-complex GN [58].

Some diseases show mixed or atypical presentations. IgA nephropathy may present with recurrent gross hematuria and mild proteinuria, which can progress to nephrotic levels [59]. Lupus nephritis and membranoproliferative GN often show both nephritic and nephrotic features, proteinuria, hematuria, and hypertension [9]. Asymptomatic urinary abnormalities, such as isolated microscopic hematuria or subnephrotic proteinuria, may indicate early IgA nephropathy, thin basement membrane disease, or other early-stage GNs [60]. Chronic glomerulonephritis represents advanced disease with progressive scarring and renal decline, often presenting as persistent proteinuria and chronic kidney dysfunction without acute nephritic signs [61]. From a clinicopathological standpoint, podocyte-predominant diseases such as minimal change, FSGS, MN, and diabetic nephropathy tend to produce nephrotic syndrome [62]. Inflammatory diseases such as post-infectious GN, IgA nephropathy, and proliferative lupus nephritis (class III/IV) are typically nephritic [63]. Crescentic GN of any cause often leads to RPGN with severe nephritic features. However, exceptions are common; MN may include microscopic hematuria; IgA nephropathy can present with heavy proteinuria; lupus nephritis class V tends to be nephrotic; and class III/IV is nephritic or mixed [64]. These clinical syndromes, summarized in Table 2, reflect the underlying pathology and are essential in guiding diagnostic and therapeutic strategies.

**Table 2:** Summary of clinical presentations, key findings, and characteristic histopathology of major glomerular diseases. MCD: Minimal Change Disease, FSGS: Focal Segmental Glomerulosclerosis, GN: Glomerulonephritis, MN: Membranous Nephropathy, HTN: Hypertension, IF: Immunofluorescence, EM: Electron Microscopy, LM: Light Microscopy, RPGN: Rapidly Progressive Glomerulonephritis, GBM: Glomerular Basement Membrane, ANCA: Anti-neutrophil Cytoplasmic Antibody, CKD: Chronic Kidney Disease, C3: Complement Component 3, DNA: Deoxyribonucleic Acid, ANA: Antinuclear Antibody

Disease	Typical Presentation	Key Features	Characteristic Pathology
Minimal Change Disease (MCD) [25]	Nephrotic syndrome (children)	Sudden selective proteinuria; steroid-responsive	LM: normal; IF: negative; EM: diffuse podocyte effacement
FSGS [26,27]	Nephrotic or subnephrotic proteinuria (adults)	Variable response to corticosteroids; approximately 40–60% achieve remission, while steroid resistance is common in certain variants (e.g., collapsing) or secondary forms; hematuria and hypertension are frequent.	LM: segmental sclerosis; IF: ±IgM/C3; EM: segmental or patchy podocyte foot process effacement
Membranous Nephropathy (MN) [28]	Nephrotic syndrome (middle-aged adults)	Heavy proteinuria; ± microscopic hematuria; risk of thrombosis	LM: thickened capillary walls; IF: granular IgG/C3; EM: subepithelial deposits
IgA Nephropathy [65]	Gross hematuria with infections; asymptomatic microhematuria	Most common primary GN; variable proteinuria	LM: mesangial proliferation; IF: mesangial IgA; EM: mesangial deposits
Post-Strep GN [66]	Acute nephritic syndrome (1–3 weeks post-infection)	Hematuria, low C3, edema, HTN	LM: endocapillary proliferation; IF: starry-sky IgG/C3; EM: subepithelial “humps”
Lupus Nephritis [67]	Variable (nephritic, nephrotic, or mixed)	+ANA, +anti-dsDNA, low complement; systemic signs	Depends on class: “full-house” IF; EM: mesangial, subendothelial, or subepithelial deposits
ANCA-Associated GN [31]	Rapidly progressive GN ± systemic vasculitis	Rapidly progressive renal failure, red blood cell casts, systemic vasculitic symptoms; +ANCA	LM: necrotizing crescentic GN; IF: pauci-immune; EM: GBM rupture, fibrin
Anti-GBM Disease [68]	RPGN ± pulmonary hemorrhage (Goodpasture’s)	+Anti-GBM antibodies; hematuria, hemoptysis	LM: crescentic GN; IF: linear IgG; EM: GBM disruption, no deposits
Diabetic GN [69]	Proteinuria in known diabetic	Slow progression; retinopathy common	LM: nodular or diffuse sclerosis; IF: non-immune; EM: thickened GBM, expanded matrix

Diagnostic techniques in renal biopsy

Renal biopsy remains the gold standard for diagnosing glomerular diseases [70]. It is evaluated using three complementary modalities: LM, IF, and EM [71]. LM involves formalin-fixed tissue stained with H&E, PAS, silver methenamine, and trichrome stains to assess glomerular architecture, cellularity, and features such as sclerosis, crescents, necrosis, or capillary wall changes [72]. PAS highlights basement membranes and mesangial matrix; silver stain outlines the GBM; and trichrome reveals fibrosis [73]. The extent of glomerular and tubulointerstitial damage, including the number of involved glomeruli and chronicity, informs prognosis [74]. IF is performed on frozen sections to detect immune deposits using antibodies against immunoglobulins (IgG, IgA, IgM), complement (C3, C1q), fibrin, albumin, and light chains [12]. The pattern and location of deposits, e.g., full-house staining in lupus, linear IgG in anti-GBM disease, or mesangial IgA in IgA nephropathy, are diagnostic clues [12]. IF can also detect deposits in vessels or tubules [12]. In cases without frozen tissue, immunohistochemistry (IHC) may be used, although with reduced sensitivity [75]. Advanced IF can also identify disease-specific antigens such as PLA2R or collagen IV chains [76]. EM offers high-resolution visualization of podocyte foot processes, GBM structure, and immune complex location. EM is essential in diagnosing diseases such as MCD (foot process effacement), Alport syndrome (GBM splitting), post-infectious GN (subepithelial humps), and fibrillary glomerulopathy (20 nm fibrils) [77]. In some conditions, EM findings are diagnostic even when LM and IF are non-specific [78]. Special stains and adjunct tools such as Congo red (for amyloid), IgG subclass staining, and viral antigen detection can refine the diagnosis [79]. In complex or atypical cases, mass spectrometry on microdissected glomeruli helps identify unknown antigens, aiding the diagnosis of infection-related or monoclonal protein-associated GN [80]. Ultimately, these diagnostic modalities are interpreted together and in the context of clinical findings. A precise and integrated pathology report synthesizes LM, IF, EM, and IHC results, offering a definitive or probable diagnosis that guides clinical management [80]. For example, a report might read: “Stage II membranous nephropathy with 70%-foot process effacement on EM, PLA2R positive, and minimal interstitial fibrosis (5%).”

Disease-specific clinicopathological correlations

FSGS and MCD: Podocytopathies

MCD and FSGS are closely related podocytopathies, and while they share overlapping pathogenic mechanisms, it remains debated whether they represent a true continuum of a single disease spectrum or distinct clinicopathologic entities [81]. MCD is more common in children, presenting with abrupt-onset nephrotic syndrome and excellent steroid responsiveness [82]. LM appears normal, with podocyte foot process effacement visible only on EM [83]. FSGS, more prevalent in adults, especially those of African or Hispanic descent, shows segmental glomerular scarring, typically in corticomedullary glomeruli [84]. The Columbia classification identifies FSGS variants: NOS, tip, perihilar, cellular, and collapsing (the most aggressive, often linked to HIV or APOL1 alleles) [85]. MCD and primary FSGS likely share a common podocyte-targeting pathogenesis involving circulating permeability factors [86,87]. Clinically, MCD features abrupt edema with preserved renal function [88]. Remission with immunosuppression is seen in 50-60% of FSGS cases, and the collapsing variant carries a poor prognosis [89]. Secondary FSGS, caused by hyperfiltration (e.g., obesity), typically shows perihilar sclerosis, hypertrophied glomeruli, and <50% foot process effacement [90]. FSGS often progresses to ESRD within a decade, while MCD has an excellent prognosis in children, though relapse is common [91]. Steroid-resistant MCD may reflect underlying genetic FSGS (e.g., NPHS2 mutations) [92].

Membranous Nephropathy (MN)

MN is a leading cause of nephrotic syndrome in adults [93]. Approximately 70-80% of primary MN cases involve autoantibodies to the PLA2R antigen on podocytes; other target antigens include THSD7A, NELL-1, Semaphorin 3B (especially in children), EXT1/EXT2 (autoantibodies seen in autoimmune diseases such as lupus), neural cell adhesion molecule 1 (NCAM1), protein tyrosine phosphatase receptor type O (PTPRO), high-temperature requirement A serine peptidase 1 (HTRA1), and others identified through advanced mass spectrometry-based antigen discovery [94]. Anti-PLA2R antibodies in serum or deposits confirm primary MN [95]. Secondary MN may be associated with lupus, hepatitis B, malignancy, or drugs, with different immune profiles [28]. Histologically, MN shows diffuse capillary wall thickening, classic spike-and-dome appearance on silver stain, and granular IgG4 and C3 deposits on IF [12]. EM reveals subepithelial deposits, initially segmental, later embedded in GBM [96]. Clinically, MN presents with proteinuria, edema, and sometimes hematuria or mild hypertension [97]. Thrombosis risk is high with serum albumin <2.5 g/dL. One-third of patients remit spontaneously, one-third remain stable, and one-third progress to ESRD within 5-10 years [98]. High anti-PLA2R titers, persistent proteinuria, and tubulointerstitial fibrosis predict poor outcomes [99]. Per KDIGO, management is risk-stratified. All patients receive optimized supportive care (RAAS blockade, BP/edema/lipid management; consider VTE prophylaxis when serum albumin is very low). Immunosuppression is reserved for moderate-to-very-high risk disease-based on a combination of persistent proteinuria, eGFR/serum creatinine trend, serum albumin, and anti-PLA2R status. First-line options include rituximab, cyclophosphamide plus glucocorticoids (Ponticelli-type), or a calcineurin-inhibitor-based regimen (often with rituximab), with serial anti-PLA2R monitoring to guide response and retreatment [100]. 

IgA Nephropathy

IgA nephropathy is the most common primary GN worldwide, especially in Asia [101]. It results from mesangial deposition of galactose-deficient polymeric IgA1 and associated autoimmune responses [102]. The classic presentation includes gross hematuria following upper respiratory infections (synpharyngitic hematuria), though asymptomatic microscopic hematuria and mild proteinuria are also common [63]. Around 20-30% present with >1 g/day proteinuria; nephrotic-range proteinuria occurs in 10%. Disease course varies: some remain stable, while others progress to ESRD. Poor prognostic factors include persistent proteinuria >1 g/day, hypertension, and tubulointerstitial fibrosis [103]. The Oxford MEST-C classification helps assess risk [104]. IF shows dominant mesangial IgA and C3 deposition, usually without C1q (unlike lupus) [105].

Secondary causes include chronic liver disease, particularly cirrhosis, and IgA vasculitis (Henoch-Schönlein purpura) [106]. The Kidney Disease: Improving Global Outcomes (KDIGO) 2021 guidelines emphasize a risk-based approach to IgA nephropathy management. All patients receive optimized supportive care, including lifestyle measures, blood pressure control (<120 mmHg systolic), and maximal renin-angiotensin-aldosterone system (RAAS) inhibition for proteinuria ≥0.5-1 g/day [107]. Sodium-glucose cotransporter 2 (SGLT2) inhibitors are now recommended for patients with CKD and preserved kidney function, regardless of diabetes status, based on evidence from EMPA-KIDNEY and DAPA-CKD trials [107]. Immunosuppressive therapy is reserved for persistent proteinuria ≥1 g/day after three to six months of optimized care or rapidly progressive disease. Systemic glucocorticoids (oral or reduced-dose regimens) remain an option, but adverse effect risk mandates careful selection [107]. Targeted-release budesonide (TRF-budesonide, Nefecon) has demonstrated proteinuria reduction and slowed eGFR decline in phase 3 trials (NefIgArd) and is emerging as a treatment option for high-risk patients [108].

Lupus Nephritis (LN)

LN affects over 50% of SLE patients and significantly contributes to morbidity [109]. The ISN/RPS system classifies LN into six classes (I-VI), with classes III/IV (proliferative) presenting with nephritic features-hematuria, hypertension, reduced GFR, and proteinuria [110]. Class V (membranous) resembles MN with preserved function but significant proteinuria. Classes I-II are usually mild [111]. Proliferative LN (classes III/IV) is associated with elevated anti-dsDNA and low C3/C4 [112]. Biopsy confirms class and guides treatment [112]. Pathology often shows “full-house” IF staining, and EM reveals multi-compartmental immune deposits [113]. Tubuloreticular inclusions may be seen [113]. Proliferative lesions (wire loops, crescents) signify active disease, while sclerosis indicates chronic damage [114]. Classes III/IV require high-dose steroids plus cytotoxic or biologic agents [115]. Class V may be treated with RAAS blockade or immunosuppressants if mixed features are present [110]. Class IV, high chronicity scores, male sex, and Black race predict worse outcomes [116].

Post-infectious Glomerulonephritis (PIGN)

Acute PIGN, especially post-streptococcal, follows throat or skin infections in children [117]. It presents with periorbital edema, cola-colored urine, and hypertension [117]. C3 levels are low but normalize over weeks [118]. Urinalysis reveals RBC casts, and anti-streptococcal antibodies are usually positive [119]. Most children recover fully, though adults may develop CKD [119]. Biopsy shows diffuse endocapillary proliferative GN with neutrophils. IF displays “starry sky” granular C3 (±IgG) [120]. EM identifies subepithelial humps, which are diagnostic and eventually resorbed [120]. Treatment is supportive [117]. Infection-related GN during active infection (e.g., staphylococcus) may have IgA-dominant IF and requires infection control, not immunosuppression. PIGN remains more common in developing regions [121].

Diabetic Nephropathy (DN)

DN is the most common global cause of ESRD, resulting from chronic hyperglycemia-induced injury [122]. Pathogenic mechanisms include glycation, oxidative stress, and RAAS activation [123]. Classically, DN progresses from glomerular hyperfiltration to microalbuminuria (30-300 mg/day) and then to overt proteinuria, nephrotic-range proteinuria, and progressive renal function decline. However, contemporary studies show that a significant proportion of patients, particularly those with type 2 diabetes, develop non-albuminuric diabetic kidney disease (NA-DKD) characterized by declining eGFR and structural changes in the absence of significant albuminuria. This phenotype is associated with prominent tubulointerstitial and vascular injury rather than glomerular lesions, highlighting that DKD is a heterogeneous entity with multiple pathophysiologic pathways [124]. Coexisting hypertension accelerates damage [125]. Early pathology shows mesangial expansion and GBM thickening; advanced DN presents with nodular glomerulosclerosis (Kimmelstiel-Wilson nodules) [126]. Arteriolosclerosis and interstitial fibrosis are common [127]. IF is typically negative, but linear IgG may be seen [127]. EM shows mesangial matrix expansion and thickened GBM [128]. The RPS 2010 classification (classes I-IV) reflects progression from GBM thickening to nodular sclerosis [129]. Prognosis worsens with uncontrolled diabetes, proteinuria, and chronic changes [130]. Management focuses on glycemic and BP control, RAAS inhibition, SGLT2 inhibitors, and endothelin receptor antagonists [131]. No specific immunotherapy exists; tubulointerstitial damage is a key prognostic marker [132].

**Figure 1:**
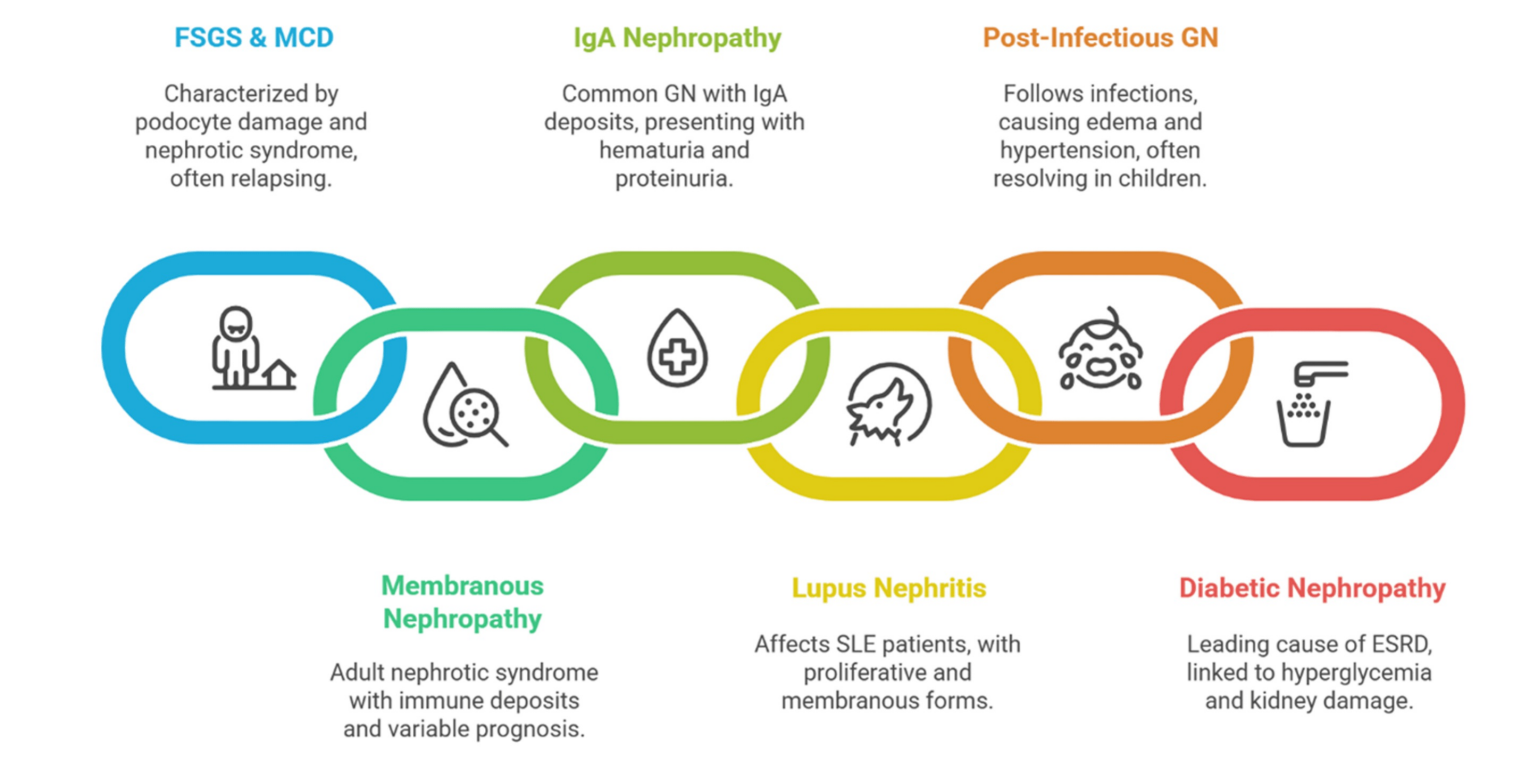
Summary of key glomerular diseases and their hallmark clinical-pathological features. FSGS: focal segmental glomerulosclerosis; MCD: minimal change disease; GN: glomerulonephritis; SLE: systemic lupus erythematosus; ESRD: end-stage renal disease The figure was created by the team and is original and not taken from any external resource. Image credits: Karis Khattab

Figure 1 illustrates a summary of key glomerular diseases and their hallmark clinical-pathological features.

Prognostic and therapeutic implications of histological patterns

Histologic features of glomerular injury are vital for prognosis and therapeutic guidance [133]. Active lesions, such as endocapillary proliferation, crescents, and necrosis, often respond to immunosuppressive therapy, while chronic changes such as global sclerosis, tubular atrophy, and interstitial fibrosis predict poor recovery, even if the underlying cause is treated [63]. Crescents, particularly cellular ones, are markers of severe glomerular injury in diseases such as ANCA-associated GN, lupus nephritis, and IgA nephropathy [134]. The percentage of affected glomeruli correlates with outcome, with >50% crescents indicating worse prognosis and the need for aggressive immunosuppression [135]. A high chronic damage burden (e.g., >40% global sclerosis or interstitial fibrosis) signifies limited potential for renal recovery [136]. In such cases (e.g., class VI lupus), immunosuppression is typically not beneficial, and care shifts to supportive or palliative approaches [137]. Tubulointerstitial fibrosis, in particular, is a strong independent predictor of long-term outcome [138]. Proteinuria is a reliable marker of disease activity and prognosis [139]. Persistent proteinuria >1 g/day is a strong and independent predictor of CKD progression across virtually all glomerular diseases. In IgA nephropathy and other proteinuric kidney disorders, persistent proteinuria frequently correlates with segmental glomerulosclerosis and tubulointerstitial fibrosis and serves as a key therapeutic target; sustained levels ≥1 g/day justify intensifying therapy, even in the setting of preserved eGFR [140]. The location of immune deposits affects both clinical presentation and treatment [140]. Subendothelial deposits (as in proliferative lupus or membranoproliferative glomerulonephritis (MPGN)) cause aggressive inflammation and require intensive immunosuppression. Subepithelial deposits (e.g., MN, lupus class V) typically present with nephrotic syndrome and may be managed more conservatively [110]. C3 glomerulopathy, with ongoing complement activation, may need targeted complement inhibitors [141]. Histologic subclassification further refines treatment strategies [141]. For example, endocapillary hypercellularity in IgA nephropathy may warrant immunosuppression, while secondary FSGS patterns (e.g., perihilar sclerosis) call for supportive therapy rather than steroids [142]. Collapsing FSGS, however, demands prompt treatment due to rapid progression [143]. Lupus class dictates therapy intensity, with classes III/IV needing induction-maintenance regimens, and class V treated based on proteinuria or mixed patterns [144]. Scoring systems, such as the MEST-C score in IgA nephropathy or NIH activity/chronicity indices in lupus, quantify lesions and predict response and outcome [145]. These tools guide individualized management and have been validated in large studies. Emerging biomarkers add prognostic value beyond histology [145]. In MN, anti-PLA2R antibody levels predict remission or relapse [146]. In ANCA vasculitis, C3 deposition or sclerotic patterns indicate poor prognosis [147]. Molecular profiling of biopsies may further enhance risk stratification and guide targeted interventions [148]. Histologic findings provide crucial diagnostic and prognostic insights that help tailor therapy; for example, lesion activity versus chronicity scores influence immunosuppression decisions, and identification of atypical features can refine diagnostic classification and guide individualized management [149]. Thus, comprehensive histopathologic evaluation remains central to precision nephrology.

Recent advances and future directions in glomerular pathology

Recent discoveries have redefined previously idiopathic glomerular diseases [150]. Key antigens such as PLA2R in MN now allow noninvasive diagnosis and disease monitoring [151]. Additional antigens (THSD7A, NELL-1, semaphorin-3B, exostosin) and proteomic techniques enable more precise subclassification, especially in atypical or infection-related GN [152]. Genetic testing is crucial in childhood steroid-resistant nephrotic syndrome and familial hematuria (e.g., NPHS1, NPHS2, WT1, COL4A mutations) [153]. APOL1 risk variants in individuals of African descent help explain the higher incidence of FSGS and HIV-associated nephropathy and are guiding transplantation and therapy decisions [154]. IgA nephropathy biomarkers such as galactose-deficient IgA1 and anti-IgA1 antibodies are under investigation [155]. Moreover, standardized systems such as the revised 2018 lupus nephritis classification and the Oxford MEST-C scoring for IgA nephropathy improve consistency and prognostication [156]. Modern reporting emphasizes capturing all pathologic findings, including mixed patterns, enhancing personalized treatment decisions [157]. Additionally, digital pathology and AI are enhancing biopsy evaluation [157]. Machine learning models, such as convolutional neural networks (CNNs), accurately classify glomerular lesions and quantify chronic damage [158]. These tools support consistency, aid early detection, and facilitate remote consultations [158]. In addition, targeted therapies are transforming treatment. Rituximab has become first-line for MN, while budesonide and sparsentan show promise in IgA nephropathy [159]. In ANCA vasculitis, rituximab and avacopan offer steroid-sparing options [160]. Lupus nephritis treatments now include agents such as voclosporin, belimumab, and obinutuzumab [161]. Moreover, advances in immunosuppressives, supportive care, and RRT have improved long-term outcomes [162]. Transplantation is effective in most glomerular diseases, though recurrence remains a concern in FSGS and IgA nephropathy [163]. Molecular monitoring (e.g., anti-dsDNA, anti-PLA2R, urinary biomarkers) enables early intervention and better disease tracking alongside histology [164]. Moreover, transcriptomic analysis of biopsy tissue is helping identify markers that predict therapy response or disease progression [165]. These novel approaches may eventually redefine treatment for refractory or relapsing glomerular diseases.

## Conclusions

In conclusion, the histopathological patterns of glomerular injury serve as a roadmap to both diagnosis and management. By classifying glomerular diseases based on morphology, etiology, and immunopathology, and correlating these with clinical syndromes, we can stratify patients for appropriate therapy and prognostication. The kidney biopsy, far from being an academic exercise, remains the cornerstone for patient-centric decisions in glomerular disease - a living example of how art (visual pathology) meets science (immunology and genetics) for optimal care. Continuing research and collaboration between clinicians and pathologists will further refine these patterns and correlations, ultimately improving outcomes for patients with glomerular diseases.

## References

[1] Pollak MR, Quaggin SE, Hoenig MP, Dworkin LD (2014). The glomerulus: the sphere of influence. Clin J Am Soc Nephrol.

[2] Menon MC, Chuang PY, He CJ (2012). The glomerular filtration barrier: components and crosstalk. Int J Nephrol.

[3] Arif E, Nihalani D (2013). Glomerular filtration barrier assembly: an insight. Postdoc J.

[4] Ding X, Cheng Z, Qian Q (2017). Intravenous fluids and acute kidney injury. Blood Purif.

[5] Daehn IS, Duffield JS (2021). The glomerular filtration barrier: a structural target for novel kidney therapies. Nat Rev Drug Discov.

[6] Imig JD, Zhao X, Elmarakby AA, Pavlov T (2022). Interactions between podocytes, mesangial cells, and glomerular endothelial cells in glomerular diseases. Front Physiol.

[7] Luyckx VA, Tonelli M, Stanifer JW (2018). The global burden of kidney disease and the sustainable development goals. Bull World Health Organ.

[8] Barutta F, Bellini S, Gruden G (2022). Mechanisms of podocyte injury and implications for diabetic nephropathy. Clin Sci (Lond).

[9] Khanna R (2011). Clinical presentation and management of glomerular diseases: hematuria, nephritic and nephrotic syndrome. Mo Med.

[10] Verma PR, Patil P (2024). Nephrotic syndrome: a review. Cureus.

[11] Gonzalez FM, Valjalo R (2025). Essential role of kidney biopsy in diagnosing glomerular diseases amidst evolving biomarkers. World J Nephrol.

[12] Messias N (2024). Immunofluorescence use and techniques in glomerular diseases: a review. Glomerular Dis.

[13] Windpessl M, Odler B, Bajema IM (2023). Glomerular diseases across lifespan: key differences in diagnostic and therapeutic approaches. Semin Nephrol.

[14] Linke A, Tiegs G, Neumann K (2022). Pathogenic T-cell responses in immune-mediated glomerulonephritis. Cells.

[15] Oates JC, Gilkeson GS (2006). The biology of nitric oxide and other reactive intermediates in systemic lupus erythematosus. Clin Immunol.

[16] Anders HJ, Kitching AR, Leung N, Romagnani P (2023). Glomerulonephritis: immunopathogenesis and immunotherapy. Nat Rev Immunol.

[17] Smith RJ, Appel GB, Blom AM (2019). C3 glomerulopathy - understanding a rare complement-driven renal disease. Nat Rev Nephrol.

[18] Jiang H, Shen Z, Zhuang J (2023). Understanding the podocyte immune responses in proteinuric kidney diseases: from pathogenesis to therapy. Front Immunol.

[19] Shankland SJ (2006). The podocyte's response to injury: role in proteinuria and glomerulosclerosis. Kidney Int.

[20] Sim JJ, Smoyer WE, Schachter AD (2024). Minimal change disease and FSGS are a spectrum of a single disease within immune-mediated nephrotic syndrome. Kidney360.

[21] Magro C, Mulvey JJ, Berlin D (2020). Complement associated microvascular injury and thrombosis in the pathogenesis of severe COVID-19 infection: a report of five cases. Transl Res.

[22] Gluhovschi C, Gadalean F, Velciov S, Nistor M, Petrica L (2023). Three diseases mediated by different immunopathologic mechanisms—ANCA-associated vasculitis, anti-glomerular basement membrane disease, and immune complex-mediated glomerulonephritis—a common clinical and histopathologic picture: rapidly progressive crescentic glomerulonephritis. Biomedicines.

[23] Mastrangelo A, Serafinelli J, Giani M, Montini G (2020). Clinical and pathophysiological insights into immunological mediated glomerular diseases in childhood. Front Pediatr.

[24] Kiran N, Sapna F, Kiran F (2023). Digital pathology: transforming diagnosis in the digital age. Cureus.

[25] Maas RJ, Nijenhuis T, van der Vlag J (2022). Minimal change disease: more than a podocytopathy?. Kidney Int Rep.

[26] Pačić A, Šenjug P, Bacalja J (2017). IgM as a novel predictor of disease progression in secondary focal segmental glomerulosclerosis. Croat Med J.

[27] Beaudreuil S, Lorenzo HK, Elias M, Nnang Obada E, Charpentier B, Durrbach A (2017). Optimal management of primary focal segmental glomerulosclerosis in adults. Int J Nephrol Renovasc Dis.

[28] Moroni G, Ponticelli C (2020). Secondary membranous nephropathy: a narrative review. Front Med (Lausanne).

[29] Sethi S, Nester CM, Smith RJ (2012). Membranoproliferative glomerulonephritis and C3 glomerulopathy: resolving the confusion. Kidney Int.

[30] Raj R, Sharma A, Barwad A (2022). KM55 in the evaluation of IgA-containing glomerular diseases. Glomerular Dis.

[31] Syed R, Rehman A, Valecha G, El-Sayegh S (2015). Pauci-immune crescentic glomerulonephritis: an ANCA-associated vasculitis. Biomed Res Int.

[32] Hommos MS, Zeng C, Liu Z (2018). Global glomerulosclerosis with nephrotic syndrome; the clinical importance of age adjustment. Kidney Int.

[33] Alsaad KO, Herzenberg AM (2007). Distinguishing diabetic nephropathy from other causes of glomerulosclerosis: an update. J Clin Pathol.

[34] Kidney International Supplements (2012). Chapter 2: general principles in the management of glomerular disease. Kidney Int Suppl (2011).

[35] Yu SM, Deoliveira M, Chung M, Lafayette R (2024). Membranoproliferative glomerulonephritis pattern of injury. Adv Kidney Dis Health.

[36] Kolev M, Horn MP, Semmo N, Nagler M (2022). Rational development and application of biomarkers in the field of autoimmunity: a conceptual framework guiding clinicians and researchers. J Transl Autoimmun.

[37] Lv J, Yang Y, Zhang H (2013). Prediction of outcomes in crescentic IgA nephropathy in a multicenter cohort study. J Am Soc Nephrol.

[38] Faucon AL, Lando S, Chrysostomou C (2025). Primary glomerular diseases and long-term adverse health outcomes: a nationwide cohort study. J Intern Med.

[39] Dattani R, McAdoo S (2019). Secondary glomerular disease. Medicine.

[40] Giordano L, Cacciola R, Barone P (2024). Autoimmune diseases and plasma cells dyscrasias: pathogenetic, molecular and prognostic correlations. Diagnostics (Basel).

[41] Weening JJ, D'Agati VD, Schwartz MM (2004). The classification of glomerulonephritis in systemic lupus erythematosus revisited. Kidney Int.

[42] Javed T, Vohra P (2012). Crescentic glomerulonephritis with anti-GBM and p-ANCA antibodies. Case Rep Nephrol.

[43] Truong L, Seshan SV (2021). Lupus nephritis: the significant contribution of electron microscopy. Glomerular Dis.

[44] Bharati J, Jhaveri KD, Salama AD, Oni L (2024). Anti-glomerular basement membrane disease: recent updates. Adv Kidney Dis Health.

[45] Cassano Cassano R, Bonadio AG, Del Giudice ML, Giannese D, Galimberti S, Buda G (2025). Light chain deposition disease: pathogenesis, clinical characteristics and treatment strategies. Ann Hematol.

[46] Sethi S, Haas M, Markowitz GS (2016). Mayo Clinic/Renal Pathology Society consensus report on pathologic classification, diagnosis, and reporting of GN. J Am Soc Nephrol.

[47] Chauhan S, Jain S, Garg N, Dixit S, Sharma S (2021). Crescents in kidney biopsy - what do they imply? A clinicopathologic study of 40 cases in a tertiary care center. J Microsc Ultrastruct.

[48] Madaio MP, Harrington JT (2001). The diagnosis of glomerular diseases: acute glomerulonephritis and the nephrotic syndrome. Arch Intern Med.

[49] Macé C, Chugh SS (2014). Nephrotic syndrome: components, connections, and angiopoietin-like 4-related therapeutics. J Am Soc Nephrol.

[50] Hebert LA, Parikh S, Prosek J, Nadasdy T, Rovin BH (2013). Differential diagnosis of glomerular disease: a systematic and inclusive approach. Am J Nephrol.

[51] Diskin CJ, Stokes TJ, Dansby LM, Carter TB, Radcliff L, Thomas SG (1999). Towards an understanding of oedema. BMJ.

[52] Parker K, Ragy O, Hamilton P, Thachil J, Kanigicherla D (2023). Thromboembolism in nephrotic syndrome: controversies and uncertainties. Res Pract Thromb Haemost.

[53] Horváth O, Szabó AJ, Reusz GS (2023). How to define and assess the clinically significant causes of hematuria in childhood. Pediatr Nephrol.

[54] Rawla P, Padala SA, Ludhwani D (2025). Poststreptococcal glomerulonephritis. StatPearls.

[55] Hashmi MS, Pandey J (2023). Nephritic syndrome. StatPearls.

[56] Saha MK, Massicotte-Azarniouch D, Reynolds ML, Mottl AK, Falk RJ, Jennette JC, Derebail VK (2022). Glomerular hematuria and the utility of urine microscopy: a review. Am J Kidney Dis.

[57] Anguiano L, Kain R, Anders HJ (2020). The glomerular crescent: triggers, evolution, resolution, and implications for therapy. Curr Opin Nephrol Hypertens.

[58] Mavratsas VC, Vu L, Yeh OL (2024). An insidious case of rapidly progressive glomerulonephritis secondary to pauci-immune crescentic glomerulonephritis. Cureus.

[59] Kim JK, Kim JH, Lee SC (2012). Clinical features and outcomes of IgA nephropathy with nephrotic syndrome. Clin J Am Soc Nephrol.

[60] Kallash M, Rheault MN (2020). Approach to persistent microscopic hematuria in children. Kidney360.

[61] Jennette JC, Falk RJ 16 - Glomerular clinicopathologic syndromes. National Kidney Foundation Primer on Kidney Diseases (Sixth Edition).

[62] Rathi M, Bhagat RL, Mukhopadhyay P (2014). Changing histologic spectrum of adult nephrotic syndrome over five decades in north India: a single center experience. Indian J Nephrol.

[63] Rajasekaran A, Julian BA, Rizk DV (2021). IgA nephropathy: an interesting autoimmune kidney disease. Am J Med Sci.

[64] Provenzano LF, Herlitz L (2018). Infection-related glomerulonephritis. National Kidney Foundation's Primer on Kidney Diseases.

[65] Filippone EJ, Gulati R, Farber JL (2024). Contemporary review of IgA nephropathy. Front Immunol.

[66] Oba Y, Mizuno H, Taneda S (2024). Anti-factor H antibody-positive C3 glomerulonephritis secondary to poststreptococcal acute glomerulonephritis with diabetic nephropathy. CEN Case Rep.

[67] Nelson MC, Rytting H, Greenbaum LA, Goldberg B (2022). Presentation of SLE after COVID vaccination in a pediatric patient. BMC Rheumatol.

[68] Sporinova B, McRae SA, Muruve DA, Fritzler MJ, Nasr SH, Chin AC, Benediktsson H (2019). A case of aggressive atypical anti-GBM disease complicated by CMV pneumonitis. BMC Nephrol.

[69] Piccoli GB, Grassi G, Cabiddu G (2015). Diabetic kidney disease: a syndrome rather than a single disease. Rev Diabet Stud.

[70] Schnuelle P (2023). Renal biopsy for diagnosis in kidney disease: indication, technique, and safety. J Clin Med.

[71] Jain S, Chauhan S, Dixit S, Garg N, Sharma S (2021). Role of direct immunofluorescence microscopy in spectrum of diffuse proliferative glomerulonephritis: a single-center study. J Microsc Ultrastruct.

[72] Agarwal SK, Sethi S, Dinda AK (2013). Basics of kidney biopsy: a nephrologist's perspective. Indian J Nephrol.

[73] Adeva-Andany MM, Carneiro-Freire N (2022). Biochemical composition of the glomerular extracellular matrix in patients with diabetic kidney disease. World J Diabetes.

[74] Hodgkins KS, Schnaper HW (2012). Tubulointerstitial injury and the progression of chronic kidney disease. Pediatr Nephrol.

[75] Janardhan KS, Jensen H, Clayton NP, Herbert RA (2018). Immunohistochemistry in investigative and toxicologic pathology. Toxicol Pathol.

[76] van de Logt AE, Fresquet M, Wetzels JF, Brenchley P (2019). The anti-PLA2R antibody in membranous nephropathy: what we know and what remains a decade after its discovery. Kidney Int.

[77] Haas M, Seshan SV, Barisoni L (2020). Consensus definitions for glomerular lesions by light and electron microscopy: recommendations from a working group of the Renal Pathology Society. Kidney Int.

[78] Abraham M, Khosroshahi A (2017). Diagnostic and treatment workup for IgG4-related disease. Expert Rev Clin Immunol.

[79] Yakupova EI, Bobyleva LG, Vikhlyantsev IM, Bobylev AG (2019). Congo Red and amyloids: history and relationship. Biosci Rep.

[80] Sethi S, Theis JD, Palma LM, Madden B (2024). From patterns to proteins: mass spectrometry comes of age in glomerular disease. J Am Soc Nephrol.

[81] Maslyennikov Y, Bărar AA, Rusu CC (2025). The spectrum of minimal change disease/focal segmental glomerulosclerosis: from pathogenesis to proteomic biomarker research. Int J Mol Sci.

[82] Roman M, Nowicki M (2024). Detailed pathophysiology of minimal change disease: insights into podocyte dysfunction, immune dysregulation, and genetic susceptibility. Int J Mol Sci.

[83] Guo Y, Ren Y, Shi S (2025). Effects of podocyte foot process effacement on kidney prognosis and response to immunosuppressive therapy in IgA nephropathy. Kidney Med.

[84] Sprangers B, Meijers B, Appel G (2016). FSGS: diagnosis and diagnostic work-up. Biomed Res Int.

[85] Rosenberg AZ, Kopp JB (2017). Focal segmental glomerulosclerosis. Clin J Am Soc Nephrol.

[86] Reiser J, Nast CC, Alachkar N (2014). Permeability factors in focal and segmental glomerulosclerosis. Adv Chronic Kidney Dis.

[87] Cravedi P, Kopp JB, Remuzzi G (2013). Recent progress in the pathophysiology and treatment of FSGS recurrence. Am J Transplant.

[88] Rout P, Hashmi MF, Baradhi KM (2024). Focal segmental glomerulosclerosis. StatPearls.

[89] Caster DJ, Magalhaes B, Pennese N (2022). Efficacy and safety of immunosuppressive therapy in primary focal segmental glomerulosclerosis: a systematic review and meta-analysis. Kidney Med.

[90] Bonilla M, Efe O, Selvaskandan H, Lerma EV, Wiegley N (2024). A review of focal segmental glomerulosclerosis classification with a focus on genetic associations. Kidney Med.

[91] Jacobs-Cachá C, Vergara A, García-Carro C (2021). Challenges in primary focal segmental glomerulosclerosis diagnosis: from the diagnostic algorithm to novel biomarkers. Clin Kidney J.

[92] Tsukaguchi H, Sudhakar A, Le TC (2002). NPHS2 mutations in late-onset focal segmental glomerulosclerosis: R229Q is a common disease-associated allele. J Clin Invest.

[93] Gupta S, Connolly J, Pepper RJ, Walsh SB, Yaqoob MM, Kleta R, Ashman N (2017). Membranous nephropathy: a retrospective observational study of membranous nephropathy in north east and central London. BMC Nephrol.

[94] Salvadori M, Tsalouchos A (2022). New antigens involved in membranous nephropathy beyond phospholipase A2 receptor. World J Nephrol.

[95] Wu X, Liu L, Guo Y, Yang L (2018). Clinical value of a serum anti-PLA2R antibody in the diagnosis and monitoring of primary membranous nephropathy in adults. Int J Nephrol Renovasc Dis.

[96] Ren KY, Hou J (2023). Characterization of membranous nephropathy with microspherular deposits. Glomerular Dis.

[97] Girisgen I, Conkar S, Bulut İK, Şen S, Mir S (2019). Membranous nephropathy in a child with crescentic glomerulonephritis: coincidence or comorbidity?. Saudi J Kidney Dis Transpl.

[98] Gyamlani G, Molnar MZ, Lu JL, Sumida K, Kalantar-Zadeh K, Kovesdy CP (2017). Association of serum albumin level and venous thromboembolic events in a large cohort of patients with nephrotic syndrome. Nephrol Dial Transplant.

[99] Krakowska-Jura K, Kler AN, Wajerowska W, Konieczny A, Banasik M (2025). Prognostic factors of proteinuria remission in primary membranous nephropathy. J Clin Med.

[100] Zand L, Fervenza FC (2023). Anti-CD20 should be the first-line treatment in high-risk membranous nephropathy. Clin Kidney J.

[101] Zaidi O, Du F, Tang Z, Bhattacharjee S, Pareja K (2024). Review on epidemiology, disease burden, and treatment patterns of IgA nephropathy in select APAC countries. BMC Nephrol.

[102] Makita Y, Suzuki H, Nakano D (2022). Glomerular deposition of galactose-deficient IgA1-containing immune complexes via glomerular endothelial cell injuries. Nephrol Dial Transplant.

[103] D'Amico G, Bazzi C (2003). Pathophysiology of proteinuria. Kidney Int.

[104] Haaskjold YL, Bjørneklett R, Bostad L, Bostad LS, Lura NG, Knoop T (2022). Utilizing the MEST score for prognostic staging in IgA nephropathy. BMC Nephrol.

[105] Sharman A, Furness P, Feehally J (2004). Distinguishing C1q nephropathy from lupus nephritis. Nephrol Dial Transplant.

[106] Saha MK, Julian BA, Novak J, Rizk DV (2018). Secondary IgA nephropathy. Kidney Int.

[107] Gomes AM, Schau B, Farinha A (2024). Emerging perspectives in the management of IgA nephropathy: a comprehensive review. Porto Biomed J.

[108] Moriyama T, Tanaka K, Iwasaki C (2014). Prognosis in IgA nephropathy: 30-year analysis of 1,012 patients at a single center in Japan. PLoS One.

[109] Arnaud L, Tektonidou MG (2020). Long-term outcomes in systemic lupus erythematosus: trends over time and major contributors. Rheumatology (Oxford).

[110] Bomback AS (2018). Nonproliferative forms of lupus nephritis: an overview. Rheum Dis Clin North Am.

[111] Arabi Z (2012). Membranous nephropathy: treatment outline and risk stratification. Avicenna J Med.

[112] Olson SW, Lee JJ, Prince LK, Baker TP, Papadopoulos P, Edison J, Abbott KC (2013). Elevated subclinical double-stranded DNA antibodies and future proliferative lupus nephritis. Clin J Am Soc Nephrol.

[113] Seshan SV, Salvatore SP (2021). Recurrent glomerular diseases in renal transplantation with focus on role of electron microscopy. Glomerular Dis.

[114] Gasparotto M, Gatto M, Binda V, Doria A, Moroni G (2020). Lupus nephritis: clinical presentations and outcomes in the 21st century. Rheumatology (Oxford).

[115] Rovin BH, Parikh SV (2014). Lupus nephritis: the evolving role of novel therapeutics. Am J Kidney Dis.

[116] Mahajan A, Amelio J, Gairy K, Kaur G, Levy RA, Roth D, Bass D (2020). Systemic lupus erythematosus, lupus nephritis and end-stage renal disease: a pragmatic review mapping disease severity and progression. Lupus.

[117] Ong LT (2022). Management and outcomes of acute post-streptococcal glomerulonephritis in children. World J Nephrol.

[118] Kakajiwala A, Bhatti T, Kaplan BS, Ruebner RL, Copelovitch L (2016). Post-streptococcal glomerulonephritis associated with atypical hemolytic uremic syndrome: to treat or not to treat with eculizumab?. Clin Kidney J.

[119] Alhamoud MA, Salloot IZ, Mohiuddin SS (2021). A comprehensive review study on glomerulonephritis associated with post-streptococcal infection. Cureus.

[120] DiFranza LT, Markowitz GS, D'Agati VD, Santoriello D (2021). Atypical infection-related glomerulonephritis with “masked” IgG-kappa crystalline hump-like deposits. Kidney Int Rep.

[121] Takayasu M, Hirayama K, Shimohata H, Kobayashi M, Koyama A (2022). Staphylococcus aureus infection-related glomerulonephritis with dominant IgA deposition. Int J Mol Sci.

[122] Lim AKh (2014). Diabetic nephropathy - complications and treatment. Int J Nephrol Renovasc Dis.

[123] Yang P, Feng J, Peng Q, Liu X, Fan Z (2019). Advanced glycation end products: potential mechanism and therapeutic target in cardiovascular complications under diabetes. Oxid Med Cell Longev.

[124] Weir MR (2004). Microalbuminuria in type 2 diabetics: an important, overlooked cardiovascular risk factor. J Clin Hypertens (Greenwich).

[125] Petrie JR, Guzik TJ, Touyz RM (2018). Diabetes, hypertension, and cardiovascular disease: clinical insights and vascular mechanisms. Can J Cardiol.

[126] Qi C, Mao X, Zhang Z, Wu H (2017). Classification and differential diagnosis of diabetic nephropathy. J Diabetes Res.

[127] Herzog EL, Mathur A, Tager AM, Feghali-Bostwick C, Schneider F, Varga J (2014). Review: interstitial lung disease associated with systemic sclerosis and idiopathic pulmonary fibrosis: how similar and distinct?. Arthritis Rheumatol.

[128] Kriz W, Löwen J, Federico G, van den Born J, Gröne E, Gröne HJ (2017). Accumulation of worn-out GBM material substantially contributes to mesangial matrix expansion in diabetic nephropathy. Am J Physiol Renal Physiol.

[129] Kim T, Kwak Y, Lee JY, Shin H, Kim JS, Yang JW, Eom M (2022). Pathological validation of the Japanese Renal Pathology Society classification and challenges in predicting renal prognosis in patients with diabetic nephropathy. Kidney Res Clin Pract.

[130] Kumar M, Dev S, Khalid MU (2023). The bidirectional link between diabetes and kidney disease: mechanisms and management. Cureus.

[131] Hannouneh ZA, Cervantes CE, Hanouneh M, Atta MG (2025). Sodium-glucose cotransporter 2 inhibitors in diabetic kidney disease and beyond. Glomerular Dis.

[132] Sahutoglu T, Perazella MA (2025). Update on acute tubulointerstitial nephritis: clinical features, immunologic insights, and diagnostic and treatment approaches. Kidney Int Rep.

[133] Haas M, Rastaldi MP, Fervenza FC (2014). Histologic classification of glomerular diseases: clinicopathologic correlations, limitations exposed by validation studies, and suggestions for modification. Kidney Int.

[134] Iwano M, Yamaguchi Y, Iwamoto T (2012). Urinary FSP1 is a biomarker of crescentic GN. J Am Soc Nephrol.

[135] Di D, Liu L, Wang Y, Yang Y, Jiang S, Li W (2023). Crescents proportions above 10% are associated with unfavorable kidney outcomes in IgA nephropathy patients with partial crescent formation. Ren Fail.

[136] Nogueira A, Pires MJ, Oliveira PA (2017). Pathophysiological mechanisms of renal fibrosis: a review of animal models and therapeutic strategies. In Vivo.

[137] Bankole AA, Nwaonu JN (2022). The shifting landscape of lupus nephritis management: a review. Cureus.

[138] Zhang X, Zhang M, Shi X (2025). Total tubulointerstitial score: a simple and effective predictor of long-term mortality and adverse renal outcomes in lupus nephritis. Lupus Sci Med.

[139] Tanihara S, Hayakawa T, Oki I (2005). Proteinuria is a prognostic marker for cardiovascular mortality: NIPPON DATA 80, 1980-1999. J Epidemiol.

[140] Satirapoj B, Chueaboonchai T, Nata N, Supasyndh O (2025). Kidney outcomes with corticosteroid treatment in IgA nephropathy according to the Oxford-MEST-C classification. Glomerular Dis.

[141] Mastellos DC, Reis ES, Ricklin D, Smith RJ, Lambris JD (2017). Complement C3-targeted therapy: replacing long-held assertions with evidence-based discovery. Trends Immunol.

[142] Chakera A, MacEwen C, Bellur SS, Chompuk LO, Lunn D, Roberts IS (2016). Prognostic value of endocapillary hypercellularity in IgA nephropathy patients with no immunosuppression. J Nephrol.

[143] Alasfar S, Matar D, Montgomery RA (2018). Rituximab and therapeutic plasma exchange in recurrent focal segmental glomerulosclerosis postkidney transplantation. Transplantation.

[144] Rovin BH, Ayoub IM, Chan TM, Liu Z-H, Mejía-Vilet JM, Floege J (2024). KDIGO 2024 Clinical Practice Guideline for the management of lupus nephritis. Kidney Int.

[145] Bajema IM, Wilhelmus S, Alpers CE (2018). Revision of the International Society of Nephrology/Renal Pathology Society classification for lupus nephritis: clarification of definitions, and modified National Institutes of Health activity and chronicity indices. Kidney Int.

[146] Rodas LM, Matas-García A, Barros X (2019). Antiphospholipase 2 receptor antibody levels to predict complete spontaneous remission in primary membranous nephropathy. Clin Kidney J.

[147] Xiao X, Ren H, Gao P (2022). What is the prognosis of ANCA-associated glomerulonephritis with immune deposition?. Ren Fail.

[148] Pandey S, Yadav P (2025). Liquid biopsy in cancer management: integrating diagnostics and clinical applications. Pract Lab Med.

[149] Bharati J, Yang Y, Sharma P, Jhaveri KD (2023). Atypical anti-glomerular basement membrane disease. Kidney Int Rep.

[150] Ebrahimi N, Mehr AP, Seethapathy H (2025). Updates on glomerular diseases: a summary of inaugural GlomCon Hawaii 2024. Glomerular Dis.

[151] Bobart SA, Han H, Tehranian S (2021). Noninvasive diagnosis of PLA2R-associated membranous nephropathy: a validation study. Clin J Am Soc Nephrol.

[152] Ronco P, Debiec H (2021). Membranous nephropathy: current understanding of various causes in light of new target antigens. Curr Opin Nephrol Hypertens.

[153] Preston R, Stuart HM, Lennon R (2019). Genetic testing in steroid-resistant nephrotic syndrome: why, who, when and how?. Pediatr Nephrol.

[154] Friedman DJ, Pollak MR (2021). APOL1 nephropathy: from genetics to clinical applications. Clin J Am Soc Nephrol.

[155] Such-Gruchot A, Mizerska-Wasiak M, Płatos E, Pańczyk-Tomaszewska M (2025). Modern biomarkers in IgA nephropathy and their potential in paediatric research. J Clin Med.

[156] Ștefan G, Alamartine E, Mariat C, Maillard N (2024). Systematic review of the link between Oxford MEST-C classification and complement activation in IgA nephropathy. Kidney Int Rep.

[157] Ahuja S, Zaheer S (2025). Advancements in pathology: digital transformation, precision medicine, and beyond. J Pathol Inform.

[158] Yamaguchi R, Kawazoe Y, Shimamoto K (2021). Glomerular classification using convolutional neural networks based on defined annotation criteria and concordance evaluation among clinicians. Kidney Int Rep.

[159] Glassock RJ (2022). IgA nephropathy: "The times they are a-changin". Glomerular Dis.

[160] van Leeuwen JR, Quartuccio L, Draibe JB, Gunnarson I, Sprangers B, Teng YK (2025). Evaluating avacopan in the treatment of ANCA-associated vasculitis: design, development and positioning of therapy. Drug Des Devel Ther.

[161] Rossi GM, Vaglio A (2025). New treatment regimens, new drugs, and new treatment goals for lupus nephritis. J Clin Med.

[162] Ragy O, Hamilton P, Pathi A, Ahmed AA, Mitra S, Kanigicherla DA (2023). Long-term safety, clinical and immunological outcomes in primary membranous nephropathy with severe renal impairment treated with cyclophosphamide and steroid-based regimen. Glomerular Dis.

[163] Uffing A, Hullekes F, Riella LV, Hogan JJ (2021). Recurrent glomerular disease after kidney transplantation: diagnostic and management dilemmas. Clin J Am Soc Nephrol.

[164] Kanigicherla D, Gummadova J, McKenzie EA (2013). Anti-PLA2R antibodies measured by ELISA predict long-term outcome in a prevalent population of patients with idiopathic membranous nephropathy. Kidney Int.

[165] Tsimberidou AM, Fountzilas E, Bleris L, Kurzrock R (2022). Transcriptomics and solid tumors: the next frontier in precision cancer medicine. Semin Cancer Biol.

